# Bacteria-Mediated Anomalous Rho GTPase Activation Alters Sperm Structure and Provokes Premature Capacitation Events: A Possible Mechanism of Infertility

**DOI:** 10.3390/ijms26083783

**Published:** 2025-04-17

**Authors:** Bárbara Rivera, Claudia Aroca, Braian González, Neftalí Guzmán, Pablo Letelier, Pamela Uribe, Miguel Fornés, Juana Valentina Villegas, Rodrigo Boguen

**Affiliations:** 1 Laboratorio de Investigación en Salud de Precisión, Departamento de Procesos Diagnósticos y Evaluación, Facultad de Ciencias de la Salud, Universidad Católica de Temuco, Temuco 4780000, Chile; barbara.rivera@uct.cl (B.R.); claudiaaroca.tm@gmail.com (C.A.); tm.braiangonzalezv@gmail.com (B.G.); nguzman@uct.cl (N.G.); pletelier@uct.cl (P.L.); 2 Departamento de Medicina Interna, Facultad de Medicina, Universidad de La Frontera, Temuco 4780000, Chile; pamela.uribe@ufrontera.cl (P.U.); juana.villegas@ufrontera.cl (J.V.V.); 3 Laboratorio de Investigación de Andrología de Mendoza, Instituto de Histología y Embriología, Departamento de Morfología y fisiología, Escuela de Medicina, Universidad Nacional de Cuyo, CONICET, Mendoza M5500, Argentina; mfornes@fcm.uncu.edu.ar

**Keywords:** cytotoxic necrotizing factor-1, human spermatozoa, Rho GTPases, acrosome reaction, *Escherichia coli*, sperm morphology

## Abstract

Male infertility is often linked to sperm quality issues; however, the mechanisms behind these alterations remain unclear in certain contexts. This study investigates the impact of anomalous Rho GTPase activation—a process triggered by bacterial toxins—on human sperm structure and function. Human spermatozoa were exposed in vitro to a Rho GTPase activator derived from *Escherichia coli* under both capacitating and non-capacitating conditions. The results showed increased RhoA GTPase activity in non-capacitating conditions, without affecting viability or mitochondrial membrane potential. However, progressive motility decreased across both conditions, while non-progressive motility and acrosome reaction rates increased. Additionally, intracellular calcium levels rose exclusively in non-capacitating conditions. Structural analysis revealed an increase in abnormal sperm morphology, particularly vacuoles in the sperm head. These findings highlight that anomalous Rho GTPase activation disrupts essential processes like motility and capacitation, which are crucial for successful fertilization. This study provides novel insights into how bacterial infections may induce sperm damage, proposing that Rho GTPase activity could serve as a biomarker for evaluating sperm quality in cases of infertility linked to urogenital infections. Understanding these mechanisms may improve diagnostic and therapeutic approaches for male infertility associated with bacterial pathogens. Human spermatozoa were exposed in vitro to a Rho GTPase activator derived from *Escherichia coli* under both capacitating and non-capacitating conditions.

## 1. Introduction

Infertility is a problem for couples, ranked fifth by the World Health Organization (WHO) in the list of moderate to severe disabilities in the population aged under 60 years [1]. It is estimated that there are 48.5 million infertile couples in the world, and around 50% of these cases are attributable to conditions of the male factor [2]. Male infertility arises from congenital, acquired, or idiopathic causes, with congenital absence of the vas deferens, varicocele, and lifestyle factors being the most common in each category, respectively [3]. However, urogenital infections represent a significant, yet often overlooked, cause. These infections, which are frequently asymptomatic and, thus, chronic, can induce persistent inflammation and oxidative stress, leading to sperm dysfunction. This dysfunction manifests as reduced sperm concentration and motility and abnormal morphology, ultimately impairing fertility [3,4].

Microorganisms of clinical importance due to sperm quality loss include uropathogenic *Escherichia coli* (UPEC) and *Chlamydia trachomatis* [5]. UPEC is responsible for 50% to 70% of complicated infections of the urinary tract [6] and is the principal microorganism isolated in prostatitis and epididymitis [7]. It has been proposed that bacteria can damage spermatozoa through the release of toxins, as is the case of the secretion of alpha-hemolysin by UPEC [8]. Other potentially damaging toxins exist, such as the UPEC cytotoxic necrotizing factor (CNF-1) and *Chlamydia trachomatis* toxin CT166. These toxins activate proteins known Rho family GTPases (Rho GTPases) in the somatic cells, leading to disruption of the actin cytoskeleton [9,10].

Rho GTPases are a group of small intercellular proteins known for their role in regulating the actin cytoskeleton, cell motility, cell polarity, vesicular movement, and the cell cycle [11]. These proteins belong to the RAS superfamily, of which RhoA, Rac, and Cdc42 are the principal members [12]. It has been described that these GTPases have a highly regulated activation and inhibition cycle, with the active form linked to GTP and the inactive form linked to GDP [11]. Rho GTPase regulation may be altered by the action of bacterial toxins; this is the case of the UPEC CNF-1 toxin, which triggers the re-ordering of the actin cytoskeleton of urogenital epithelium cells in order to enter and colonize it [13]. It has also been described that CNF-1 causes deamination of the Rho GTPases, promoting their constant activation; in this way, UPEC can regulate the organization of the stress fibers of the actin cytoskeleton in somatic cells [9]. Although the spermatozoa eliminate remnants of the cytoplasm during its maturation, the presence of Rho GTPases—such as RhoA, Rac, and Cdc42—in the male gamete has been reported [14]. The specific function of Rac1 in human spermatozoa during sperm capacitation has not been shown; however, it has been observed that in guinea pigs, it plays an important role in actin polymerization during sperm capacitation and possibly an indirect role in the acrosome reaction (AR) [15].

In the human spermatozoa, actin is found principally in the acrosome and in the fibrous sheath that surrounds the axoneme and the dense external fibers of the flagellum [16]. The presence of actin in these zones is fundamental during human fertilization as it is involved in the events that succeed ejaculation, such as capacitation and AR [17]. During sperm capacitation, the amount of polymerized actin filaments in the sperm flagellum and head increases; then, it decreases in the sperm head only during AR, probably due to the loss of acrosome [18]. In addition, sperm capacitation must be an ordered process in order to penetrate the zona pellucida and induce acrosome reaction and, finally, oocyte fusion [19]. Sperm capacitation is also associated with an increase in intracellular calcium ([Ca^2+^]i) due to the presence of progesterone [20]; this allows calcium to enter and bind to bicarbonate (HCO_3_^−^) using specific channels like CatSper, thus promoting AR [21,22].

The toxins produced by bacteria that colonize the urogenital tract, such as UPEC CNF-1 [13] and *Chlamydia trachomatis* CT166 [10,23], activate Rho GTPases, leading to actin modifications and alterations in the actin cytoskeleton of somatic cells [9]. Rho GTPases are present in human spermatozoa [14]; however, the direct effects of the abnormal activation of Rho GTPases by toxins of urogenital pathogens on sperm function and structure have not been described. The aim of this work was to determine the effect of anomalous Rho GTPase activation on the structure and function of human spermatozoa.

## 2. Results

### 2.1. RhoA GTPase Activity

**Figure 1:**
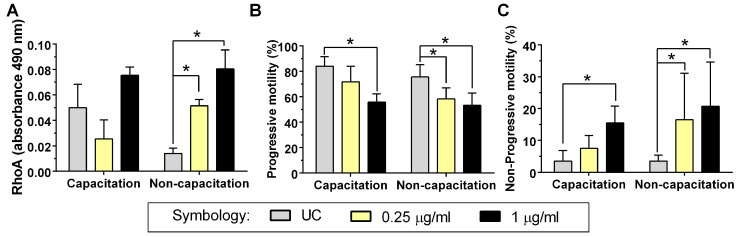
Rho Activator II effect on RhoA activity and sperm motility. Two experimental conditions were examined: a capacitation condition and a non-capacitation condition. Spermatozoa were incubated with 0.25 μg/mL and 1 μg/mL Rho Activator II for 2 h at 37 °C. The figure represents four independent samples, each with technical duplicates plus the standard deviation bar. (**A**) RhoA GTPases activity was determined by immunoassay, where the absorbance at 490 nm is proportional to RhoA activity. (**B**) Results obtained from progressive sperm motility. (**C**) Results obtained from non-progressive sperm motility. Abbreviations: UC, untreated control group without Rho Activator II; *, *p* < 0.05 compared to the UC.

An analysis of RhoA GTPase activity under capacitation conditions revealed no statistically significant changes in RhoA activity. In the non-capacitation condition, on the other hand, it was found that its activity increased significantly after 2 h of incubation with both 0.25 μg/mL and 1 μg/mL of Rho Activator II (RAII) compared to the UC group (*p* < 0.05) (Figure 1A). This indicates that RAII activates RhoA GTPase only in a non-capacitation condition. This difference could be associated with the fact that the RhoA activity of the UC was higher in the capacitation condition than in the non-capacitation condition (*p* < 0.05).

### 2.2. Viability and Mitochondrial Membrane Potential

The spermatozoa of the UC group incubated in the capacitation condition had a viability of 89.6 ± 9.5%, while for those exposed to 0.25 μg/mL and 1 μg/mL of RAII, the viability was 84.9 ± 11.4% and 87.0 ± 7.6%, respectively (*p* > 0.05). In the case of the spermatozoa in the non-capacitation condition, the viability of the UC was 88.8 ± 5.4%, while with 0.25 μg/mL and 1 μg/mL of RAII, it was 89.1 ± 4.7% and 88.2 ± 6.3%, respectively (*p* > 0.05). This shows that RAII does not alter sperm viability.

When the percentage of spermatozoa with high mitochondrial membrane potential (MMP) was evaluated in the capacitation condition, the value of the UC group was 82.0 ± 18.8%, while with 0.25 μg/mL of RAII, this value was 85.1 ± 8.4% (*p* > 0.05), and with 1 μg/mL of the activator, it was 80.7 ± 14.1% (*p* > 0.05). In the non-capacitation condition, the percentage of spermatozoa with high MMP was 81.5 ± 5.7%, while under exposure to 0.25 μg/mL and 1 μg/mL of RAII, these values were 89.8 ± 4.5% and 82.2 ± 5.2%, respectively (*p* > 0.05). Thus, there was no alteration in sperm mitochondrial activity.

### 2.3. Sperm Motility

The spermatozoa in the capacitation condition presented a significant reduction in the percentage of progressive motility (PM) compared to the UC group only under exposure to 1 μg/mL of RAII (*p* < 0.05). The spermatozoa in the non-capacitation condition presented a significant reduction in PM with both 0.25 μg/mL and 1 μg/mL of RAII (Figure 1B). When the Non-Progressive motility (NP) motility pattern was analyzed, it was shown that in the capacitation condition, there was a significant increase only with 1 μg/mL of Rho GTPase activator; however, in the non-capacitation condition, the number of NP spermatozoa increased with both concentrations of RAII (Figure 1C). The percentage of immobile (IM) spermatozoa in the capacitation condition of the UC group was 12 ± 5.4%; with 0.25 μg/mL of RAII, it was 20.8 ± 8.6% (*p* > 0.05), and with 1 μg/mL, it was 28.8 ± 5.8% (*p* < 0.05). The percentage of IM spermatozoa in the non-capacitation condition was 21 ± 10.7% for the UC group, 21 ± 12.4% with 0.25 μg/mL (*p* > 0.05), and 26 ± 13% with 1 μg/mL of RAII (*p* > 0.05). This indicates that the predominant effect of abnormal activation of Rho GTPases is on the progressivity of sperm movement; in capacitation conditions only, RAII can immobilize spermatozoa.

### 2.4. Acrosome Reaction

**Figure 2:**
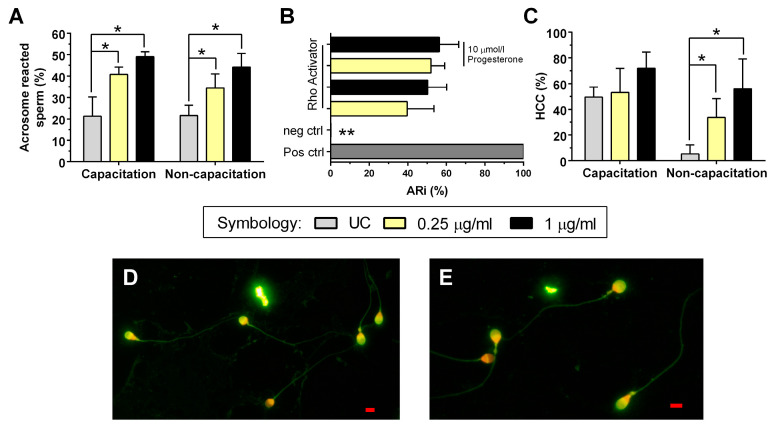
Rho Activator II effect on acrosome reaction and intracellular calcium content of spermatozoa. The figure represents four independent samples, each with technical duplicates plus the standard deviation bar. (**A**) Sperm AR measure after being incubated with 0.25 μg/mL and 1 μg/mL of Rho Activator II for 2 h at 37 °C in capacitation and non-capacitation conditions. (**B**) The sperm ARi was determined after 3 h of incubation in HTF with 5% BSA and then exposed to Rho Activator II in the presence or absence of progesterone. A negative control of sperm in the same initial medium and a positive control, to which only progesterone was added, were used. The ARi was calculated by subtracting AR spermatozoa of the negative control from experimental groups and expressed as a percentage in relation to AR spermatozoa from the positive control. (**C**) Percentage of spermatozoa with high calcium content (HCC), which were also incubated with 0.25 μg/mL and 1 μg/mL of Rho Activator II for 2 h at 37 °C in capacitated and non-capacitated conditions. (**D**) Representative image of spermatozoa staining with PSA-FITC under fluorescence microscopy (1000×) of the UC in the capacitation condition. (**E**) Representative image of spermatozoa staining with PSA-FITC under fluorescence microscopy (1000×) of the UC in the non-capacitation condition. The red line at the bottom of pictures D and E represents a 10 μm scale bar. Abbreviations: AR, acrosome reaction; HTF, human tubal fluid; BSA, bovine serum albumin; UC, untreated control without Rho Activator II; neg ctrl, negative control; Post ctrl, positive control; *, *p* < 0.05 compared to their UC group; and **, *p* < 0.05 for all groups with Rho Activator II compared to the negative control.

The analysis of the AR data obtained in the experiment shows that spermatozoa incubated in either the capacitation condition or non-capacitation condition and exposed to 0.25 μg/mL and 1 μg/mL of Rho GTPase activator presented a statistically significant increase (*p* < 0.05) in the percentage of spermatozoa with AR (Figure 2A).

These results demonstrate that AR can likewise be induced in spermatozoa in the non-capacitation condition by exposing them to RAII; to exclude the possibility that this increase was spontaneous, the result was confirmed by the functional ARi test, in which a statistically significant increase was observed in the different groups with RAII compared to the negative control of this test (*p* < 0.05). There were no observed statistical differences in the AR index (ARi) when exposed to the Rho GTPase activator in the presence or absence of 10 μmol/l of progesterone. Also, the effect of RAII on the ARi was not significant compared to the positive control for AR induction (Figure 2B). This shows that RAII does in fact induce AR in spermatozoa, in both capacitation and non-capacitation conditions.

### 2.5. Intracellular Calcium

There were no statistically significant differences in spermatozoa with HCC in the capacitation condition when exposed to the Rho GTPase activator (*p* > 0.05) (Figure 2C). The probable reason why no statistically significant differences were observed in this condition is that the UC presented 49.4 ± 7.9% of spermatozoa with HCC, while in the non-capacitation condition, the percentage of HCC in the UC was 5.4 ± 7%, which is statistically significant (*p* < 0.05). On the other hand, in the non-capacitation condition, the percentage of spermatozoa with HCC was greater with both concentrations of RAII compared to the UC (*p* < 0.05), which demonstrates that the activation of Rho GTPase also increases the calcium content in spermatozoa in the absence of capacitation stimuli like albumin and progesterone.

### 2.6. Sperm Morphology

**Figure 3:**
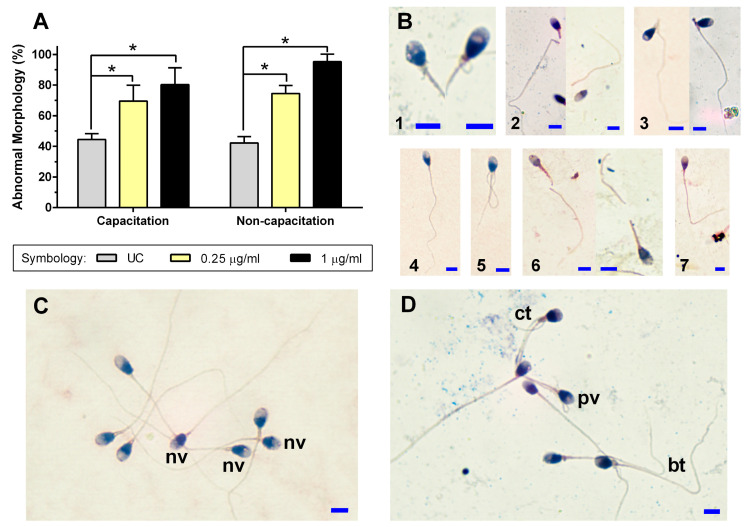
Rho Activator II effect on sperm morphology. Two experimental conditions were examined: a capacitation condition and a non-capacitation condition. Spermatozoa were incubated with 0.25 μg/mL and 1 μg/mL Rho Activator II for 2 h at 37 °C. (**A**) Percentage of spermatozoa with abnormal morphology. The figure represents four independent samples, each with technical duplicates plus the standard deviation bar. (**B**) Images of commonly observed sperm morphological abnormalities that would be associated with polymerized actin changes: 1. vacuolated head; 2. broken neck; 3. bent neck; 4. z-shaped tail; 5. coiled tail; 6. cut tail; and 7. bent tail. (**C**) Representative group of spermatozoa in the absence of Rho Activator II. It highlights the presence of vacuoles considered normal (nv). (**D**) Representative group of spermatozoa in the presence of Rho Activator II. The increased presence of morphological abnormalities such as pathological vacuoles (pv), coiled tail (ct), and bent tail (bt) compared to spermatozoa in (**C**) is highlighted. The blue line at the bottom of each picture represents a 10 μm scale bar. Abbreviations: UC, untreated group without Rho Activator II; *, *p* < 0.05 compared to their UC group.

The analysis of sperm morphology demonstrates a significant increase (*p* < 0.05) in spermatozoa with abnormal morphology after exposure to 0.25 μg/mL and 1 μg/mL of RAII in both capacitation and non-capacitation conditions compared to their UC without RAII (Figure 3A). An analysis of the principal morphological defects found in the sperm structure that might be attributable to changes in the actin cytoskeleton showed the presence of pathological vacuoles in the head (Figure 3B) after exposure to 0.25 μg/mL and 1 μg/mL of Rho GTPase activator, in contrast to the UC (*p* < 0.05) (Table 1).

There was also a significant increase in the presence of broken flagella (Figure 3C) in spermatozoa in the non-capacitation condition exposed to 0.25 μg/mL of RAII (Table 1). Finally, a predominant increase was observed in other abnormal morphologies in both the capacitation and non-capacitation conditions, such as curled flagella and z-shaped flagella, as well as a diminution in spermatozoa with a bent mid-piece; however, these differences were not statistically significant (*p* > 0.05).

## 3. Discussion

In the non-capacitation condition, a significant increase in RhoA activity was shown with both concentrations of RAII used; this shows that the activator derived from the UPEC CNF-1 toxin does indeed activate these proteins, which occur in somatic cells [9]. In contrast, such changes were not observed in the capacitation condition; this may be because the base activity of RhoA was higher than in the non-capacitation condition (Figure 1A), masking the activator effect of RAII in the capacitation condition. In addition, Rho GTPase activation in capacitation has been observed in the spermatozoa of other animal species [24,25].

A previous work reported that an alteration in the MMP affects the seminal routine parameters [8]; however, in our work, the MMP was unaltered. This was probably because RAII does not provoke changes in specific mitochondrial GTPases like Miro, which play a fundamental role in the mitochondrial activity of somatic cells in other types of pathologies [26].

In terms of motility, it was shown that both concentrations of RAII reduced sperm PM in both experimental conditions. Sperm motility after capacitation is characterized by the hyperactivation of flagellar movement to ensure beat movement [27]. This suggests that the abnormal activation of Rho GTPases may disrupt the machinery responsible for flagellar movement as the spermatozoa must reorganize the cytoskeleton and polymerize actin filaments in a coordinated manner during capacitation [17]. This agrees with our observations in the non-capacitation condition: the analysis of the results associated with sperm movement showed that PM diminished, accompanied by an increase in the number of spermatozoa with NP motility but no increase in the percentage of IM spermatozoa. This shows that RAII alters the progressivity of movement in non-capacitation conditions.

Under capacitation conditions, a higher concentration of RAII was observed to alter PM, exerting an immobilizing effect on human spermatozoa. Sperm hyperactivation is dependent on ion channels and transporter channels, as well as actin cytoskeleton dynamic and associated protein complexes [28]; the alteration or regulation of any of these will cause a diminution or loss of sperm motility. This will alter fertility [29] since any alteration in flagellar movement will reduce the fertilizing capacity of the spermatozoa [18].

During capacitation, the spermatozoa initiate processes that will finally enable AR [30]. In this research, we evaluated the effect of RAII on AR in capacitation and non-capacitation conditions. AR increased in both conditions; however, in non-capacitation conditions, the spermatozoa lacked physiological stimuli to trigger AR, from which it may be suspected that the increase in AR may be spontaneous [31]. For this reason, a functional test was applied to measure the ARi [32]. This test confirmed that RAII can induce AR in the spermatozoa directly, both in the presence and absence of progesterone.

The non-capacitation condition was established by reducing the HCO_3_^−^ and excluding progesterone [28], both of which are essential for sperm capacitation [33]. This implies that it is highly probable that the AR observed in the non-capacitation condition was solely due to an effect of Rho GTPase activator and its influence on actin filament polymerization because it has been reported that these filaments are involved in the AR process [34]. It has also been reported that *Escherichia coli* inhibits the AR induced in vitro [30]; however, a different protocol for inducing AR was used in this work, and, therefore, its results cannot be compared to ours. Another possible explanation for this discrepancy might be that the *Escherichia coli* strain used by el-Mulla et al., 1996 [30] does not produce CNF-1; it may then be hypothesized that it contains another factor or toxin capable of inhibiting AR.

During sperm capacitation, the spermatozoon internalizes calcium to trigger AR [35]. According to Boisen et al., 2021 [36], the spermatozoon must be in contact with progesterone as this will allow calcium and bicarbonate to enter the cell together. This statement disagrees with our observations since the non-capacitation condition presented an increase in the number of spermatozoa with HCC after exposure to RAII. One possible explanation is that other calcium channels exist in the spermatozoa, such as TRPV4, which is voltage-dependent and does not require the presence of progesterone [37]. In somatic cells, it has been observed that a TRPV4-mediated calcium influx leads to actin reorganization and cell migration via the pathway triggered by RhoA, which is associated with cancer metastasis [38]; thus, in the non-capacitation condition, RAII might allow calcium to enter the spermatozoa through the action of TRPV4.

According to our results for sperm morphology, RAII in vitro causes an increase in the appearance of abnormal morphologies in the spermatozoa, particularly the appearance of vacuoles in the head in both experimental conditions. In human spermatozoa, it has been observed that the presence of vacuoles shows a negative correlation with sperm motility [39], which agrees with our results. These vacuoles could result from a pathological activation of Rho GTPases because factors like an incubation temperature of 37 °C or oxidative stress do not produce vacuoles in spermatozoa [40]. Furthermore, the presence of vacuoles shows a negative correlation with fertility, pregnancy, and implantation, making it a useful marker for predicting sperm function [41]. A possible explanation for vacuole formation is that in somatic cells, the bacteria activate Rho GTPases, then, the actin filaments cause cytoplasmic prolongations, and finally, it is introduced into the cells through a vacuole [42]. According to morphological criteria, a spermatozoon can have up to two small vacuoles in the acrosomal region; anything in excess of this, in quantity, size, or location, is considered an abnormal vacuole [43]. This agrees with our observations as the vacuoles identified are considered pathological.

Other common morphological alterations to the flagellum were curled and broken flagella. The latter presented a significant increase, which correlates with the diminished PM of the spermatozoa. The morphological changes analyzed may possibly be attributed to the abnormal activation of Rho GTPases, which may modify the activation of the actin filaments of the cytoskeleton [44]. This is important because it has been reported that when the distribution of F-actin is altered, morphological changes occur in the somatic cells [45]. It is more important in the spermatozoa because the actin around the flagellum also plays a major role in regulating sperm motility [46].

Also, these findings suggest that bacterial infections secreting Rho GTPase-activating toxins, such as UPEC and *C. trachomatis*, could contribute to male infertility by impairing sperm functionality and structure. Measuring Rho GTPase activity may offer a novel biomarker for sperm damage in men with urogenital infections. Moreover, targeting Rho GTPase pathways pharmacologically could represent a therapeutic avenue to mitigate infection-induced infertility.

## 4. Materials and Methods

### 4.1. Ethics Statement

This study was approved by the Ethics Committee of the Universidad Católica de Temuco (File N° 60/20) and performed according to the ethical norms of the Declaration of Helsinki for medical research. Informed consent was submitted by all subjects when they were enrolled.

### 4.2. Sperm Collection and Selection

The semen sample was obtained from healthy-looking male donors aged over 18 years by masturbation after sexual abstinence of between 2 and 7 days. Through a brief interview before sample donation, men with chronic diseases were excluded. The men selected were normozoospermic according to the WHO’s criteria [43].

To ensure that spermatozoa of high motility and viability were used, sperm selection was performed on all the semen samples using the swim-up method [8]. Each semen sample was divided into two groups for sperm selection: the group to be used for the capacitation condition, using a human tubal fluid (HTF) medium [47], and the group to be used for the non-capacitation condition, using a modification of the HTF medium, with the bicarbonate concentration HCO_3_^−^ reduced to 4 mmol/L [48]. Finally, the selected spermatozoa concentration was determined by count in a Neubauer chamber.

### 4.3. Experimental Conditions and Activation of Rho GTPases

Two experimental conditions were used: capacitation and non-capacitation. For the capacitation condition, the HTF medium [47] was used, supplemented with bovine serum albumin (BSA) at 5% (Sigma-Aldrich, Saint Louis, MO, USA) and progesterone at 10 mmol/L (Sigma-Aldrich, Saint Louis, MO, USA) [49]. The non-capacitation condition used an HTF medium with HCO_3_^−^ reduced to 4 mmol/L [48].

The activation of Rho GTPases was induced by Rho Activator II (RAII; Cytoskeleton Inc., Denver, CO, USA), produced from UPEC CNF-1 toxin. The Rho GTPase activator was reconstituted and handled according to the manufacturer’s recommendations, at concentrations of 0.25 μg/mL and 1 μg/mL based on its ability to induce actin stress fibers.

### 4.4. Experimental Design

The final concentration of the spermatozoa selected was adjusted to 1 × 10^6^/mL for both the capacitation and the non-capacitation groups. In both conditions, the spermatozoa were exposed to two concentrations of RAII. Furthermore, for both groups, a basal control without RAII was added, called the untreated control group (UC). All the experimental conditions were incubated for 2 h at 37 °C; after that, the RhoA GTPase activity, viability, MMP, motility, AR, [Ca^2+^]i, and morphology of the spermatozoa were measured.

#### 4.4.1. Measuring RhoA GTPase Activity

RhoA activity was measured using the RhoA G-LISA Activation Assay Kit (Cytoskeleton Inc., Denver, CO, USA). In this immunoassay, the microplate wells contained a Rho GTP-binding molecule, which is detected by an antibody specific to RhoA. This was performed following the manufacturer’s instructions, using an absorbance of 490 nm, which is proportional to the RhoA activity.

#### 4.4.2. Evaluation of Viability and Mitochondrial Membrane Potential

To test that we were working with spermatozoa that were alive and that presented high mitochondrial activity, the viability and the MMP were determined with two stains: tetramethylrodamine methyl ester (TMRM) and SYTOX green. MMP is a good marker of sperm function due to its high correlation with human sperm integrity [50]. The TMRM fluorescence signal detected is proportional to the MMP. SYTOX green is a vital stain that emits green fluorescence. We used the protocol of Uribe et al., 2020 [51], with some modifications appropriate to our experimental conditions. Briefly, 1 × 10^6^/mL human spermatozoa suspended in 600 nmol/L of TMRM and 50 nmol/L of SYTOX green were incubated for 30 min at 37 °C. The spermatozoa were then washed with PBS and resuspended in the same medium for reading by a flow cytometer (BD FACSquanto II, Becton, Dickinson and Company, Franklin Lakes, NJ, USA). SYTOX green was read in the FITC channel corresponding to a 530/30 nm filter, and TMRM was read in the PE channel, which has a 585/42 nm filter. The values were expressed as percentages of live spermatozoa (SYTOX green negative) and as percentages of spermatozoa with high MMP, according to the internally controlled cut-off points.

#### 4.4.3. Measuring of Sperm Motility

The spermatozoa of the experimental conditions were analyzed by an optical microscope (Olympus CX33, Tokyo, Japan), with 400× magnification. The measurements were taken under strict controls of objectivity and rigor, following the WHO’s recommendations [43], and the results were expressed as percentages of spermatozoa with progressive motility (PM), non-progressive motility (NP) and immobile (IM).

#### 4.4.4. Evaluation of the Acrosome Reaction (AR) and Calculation of the AR Index (ARi)

To determine AR in spermatozoa, we used *Pisum Sativum Agglutinin* (PSA) lectin conjugated with fluorescein isothiocyanate (FITC). PSA-FITC is specific to D-glucose and α-D-mannose present in spermatozoa, especially in the acrosomal membranes. When AR occurs, this segment disappears; so, the marker only indicates the residual acrosomal region present in the equatorial zone of the sperm head. AR was evaluated at the end of the experimental design; based on the results obtained, the acrosome reaction index (ARi) was determined by a functional test using another experimental design [31]. The same staining protocol was used for both tests to determine AR.

The PSA-FITC staining protocol was performed by placing fractions of spermatozoa on a slide at a concentration of 1 × 10^6^/mL, in duplicate, which were dried at ambient temperature; they were then fixed and permeabilized using methanol for at least 15 min until evaporation. Then, 25 µg/mL of PSA-FITC (Sigma-Aldrich, Saint Louis, MO, USA) was added, and the sample was incubated in darkness for 30 min. The excess stain was eliminated with distilled water. The contrast medium used was propidium iodide (PI) at 100 µmol/L (Sigma-Aldrich, Saint Louis, MO, USA), which binds to the DNA and emits orange-red fluorescence. After the excess stain had been eliminated with distilled water, the sample was observed by fluorescence microscopy (Leica DM750, Wetzlar, Germany) at 1000× using an excitation light filter of 488 nm. The results were expressed as the percentage of spermatozoa with reacted acrosome, out of 200 cells counted.

To confirm whether RAII in fact induces AR and that this does not occur spontaneously, the ARi was determined by a functional test, as described in [31] and adapted to our conditions. Briefly, 1 × 10^6^/mL selected spermatozoa were incubated for 3 h in HTF supplemented with BSA at 5% to induce capacitation. After incubation, 0.25 μg/mL and 1 μg/mL of RAII were added to the spermatozoa in the presence or absence of 10 μmol/L of progesterone (Sigma-Aldrich, Saint Louis, MO, USA). As a positive control of the ARi, 10 μmol/L of progesterone was added to only one group of spermatozoa, and spermatozoa for the negative control group were kept in the initial HTF medium supplemented with BSA 5%. Both the experimental and control groups were incubated for 15 min at 37 °C and then stained with PSA-FITC following the staining protocol described above. The results were expressed as the ARi, subtracting the value of spermatozoa with AR from the negative control at each value obtained in the experimental groups, expressed as a percentage of the spermatozoa with AR in the positive control.

#### 4.4.5. Measurement of Intracellular Calcium

The [Ca^2+^]i was measured by FLUO-4 staining. This staining technique uses an acetoxymethyl ester (AM) group, which allows FLUO-4 to enter cells under metabolization and prevent it from escaping; thus, it can only bind to [Ca^2+^]i. The spermatozoa were incubated with 200 nmol/L of FLUO-4 AM for 30 min at 37 °C and then washed with PBS and centrifuged at 500× *g* for 5 min to eliminate the supernatant; the pellet was resuspended. The stained spermatozoa were read by fluorescence microscopy (Leica DM750, Wetzlar, Germany), using an excitation light filter of 488 nm. The spermatozoa with high intracellular calcium content (HCC) were distinguished from the cells with low intracellular calcium content (LCC), giving the percentage of spermatozoa with HCC on the basis of 200 spermatozoa per plate.

#### 4.4.6. Sperm Morphology Analysis

The sperm morphology was determined using Shorr staining. Before staining, the spermatozoa of the different experimental conditions were deposited on a slide. The recommended fixing and staining protocol was then performed [43]. Briefly, air-dried smears were fixed in a solution of acetic ethanol for one hour. Fixed sperm were then stained sequentially with Harris hematoxylin, ammoniacal ethanol, and Shorr stain. Following staining, slides were dehydrated through a graded series of ethanol concentrations and allowed to air-dry. Two hundred spermatozoa were individually assessed by optical microscopy at 1000× magnification (Olympus CX31, Tokyo, Japan). Normal and abnormal sperm forms were distinguished and counted. Measurements of each slide were performed in duplicate. The abnormal spermatozoa were then classified based on major morphological defects in sperm structure, which may be attributable to polymerized actin changes. These defects included heads with anomalous vacuoles, broken or bent mid-pieces, and broken, curled, bent, or Z-shaped flagella.

### 4.5. Statistical Analysis

The experimental design was performed with 4 biological replicates on different days and with 2 technical replicates for each variable measured. To detect differences, first, the values were log-transformed, and then, a two-way ANOVA was applied. The differences between the UC and the experimental groups were assessed using Bonferroni’s multiple comparison test. The ARi was analyzed by a one-way ANOVA, comparing all the results with one another using Bonferroni’s multiple comparison test. The results were considered statistically significant at *p*-values less than 0.05 (*p* < 0.05). The analyses and graphs were developed with the GraphPad Prism 6 statistics software (San Diego, CA, USA).

## 5. Conclusions

In conclusion, this study establishes that anomalous Rho GTPase activation—potentially triggered by bacterial toxins—disrupts critical aspects of sperm function and morphology. Key findings include reduced progressive motility, premature acrosome reaction, and increased pathological vacuole formation in the sperm head. These effects are likely mediated through the dysregulation of actin cytoskeleton dynamics, emphasizing the role of Rho GTPases in maintaining sperm functionality. These results highlight the potential impact of urogenital infections on male fertility, proposing that Rho GTPase activity could serve as a biomarker for sperm damage. Further research is warranted to explore therapeutic strategies targeting Rho GTPase pathways and to validate these findings in clinical settings. This work provides a molecular framework for understanding infection-induced male infertility and underscores the importance of integrating molecular diagnostics into reproductive health assessments.

## Figures and Tables

**Table 1:** Abnormal morphology of human spermatozoa subjected to RAII.

	Capacitation Condition			Non-Capacitation Condition		
Abnormal Morphology		RAII			RAII	
	UC (%)	0.25 μg/mL (%)	1 μg/mL (%)	UC (%)	0.25 μg/mL (%)	1 μg/mL (%)
Vacuolated head	0.0 ± 0.0	10.5 ± 3.4 ^a^	6.0 ± 6.4 ^a^	0.0 ± 0.0	14.3 ± 4.9 ^a^	13.0 ± 5.9 ^a^
Cut neck	0.0 ± 0.0	3.8 ± 7.5	0.0 ± 0.0	0.0 ± 0.0	0.8 ± 1.5	0.0 ± 0.0
Bent neck	10.8 ± 15.6	2.3 ± 4.5	9.8 ± 11.3	14.8 ± 23.9	6.5 ± 6.0	2.0 ± 2.4
Cut tail	10.0 ± 9.1	8.8 ± 4.9	5.5 ± 3.9	1.8 ± 3.5	11.0 ± 8.9 ^a^	5.5 ± 6.6
Coiled tail	42.5 ± 19.7	50.0 ± 13.5	36.0 ± 26.2	36.3 ± 32.0	36.8 ± 24.0	37.8 ± 12.6
Bent tail	1.8 ± 3.5	7.0 ± 12.1	0.0 ± 0.0	0.0 ± 0.0	0.0 ± 0.0	0.8 ± 1.5
Z-shaped tail	9.3 ± 10.8	0.0 ± 0.0	7.8 ± 9.7	6.8 ± 9.4	15.8 ± 18.6	32.0 ± 34.1

Values are presented as mean ± standard deviation. Abbreviations: RAII, Rho Activator II; UC, untreated control; and ^a^, *p*-value less than 0.05 compared to the UC.

## Data Availability

The data presented in this study are available upon request from the corresponding author due to ethical restrictions.
